# Neural pathway prediction based on multi-neuron spike train data

**DOI:** 10.1186/1471-2202-15-S1-P6

**Published:** 2014-07-21

**Authors:** Yi Zeng, Tielin Zhang, Bo Xu

**Affiliations:** 1 Institute of Automation, Chinese Academy of Sciences, Beijing, China; 2 University of Chinese Academy of Sciences, Beijing, China

## 

The finding of Neural Pathway is typically based on brain slicing and reconstruction with nanoscale imaging. Nevertheless, this method is not applicable when nanoscale imaging is not available. We propose that multi-neuron spike train data can be used as an alternative source to predict the neural pathways, and here we discuss two concrete strategies for neural pathway prediction based on this kind of data.

Multi-neuron spike train data is composed of spike trains from multiple neurons recorded in the same time interval. The data typically shows whether a specific neuron generates a spike or not at a specific time. Two strategies are proposed for neural pathway prediction: (1) The time-ordered strategy: Synapses exist between neurons that generate spikes at the two neighborhood time points (the time point N and N-1). (2) The spike co-occurrence strategy: Synapses exist between neurons that fire together at the same time point. This strategy is consistent with the Hebb’s law “Cells that fire together, wire together”.

Data of the rat hippocampus CA3 pyramidal cell layer based on functional Multineuron Calcium Imaging (fMCI) is used for neural pathway prediction (Including 8 datasets, and each of them records spike activities for 62 to 226 neurons. The datasets were imaged with the frequency of 10Hz [1,2]) using the upper two proposed strategies.

We validate the accuracy of the neural pathway prediction strategies based on the following steps. The overall prediction accuracy is an average value based on the 8 dataset. For each of the dataset: (1) Equally divide one dataset as 20 sub datasets according to the time intervals (The sub datasets are denoted as S_1_,…,S_20_). Select the first 80% of the sub datasets (S_1_,…,S_16_) for neural pathway construction, and validate the pathway using the rest of the 20% sub dataset (Here we assume that if the predicted pathway is correct, it should cover the neuronal connections based on the rest of the 20% sub dataset). (2) Select another 80% of the sub datasets for neural pathway construction, and the rest for validation, and repeat this step until all the sub datasets have been selected for validation. (3) The prediction accuracy is the average value of the 20 predictions.

**Figure 1:**
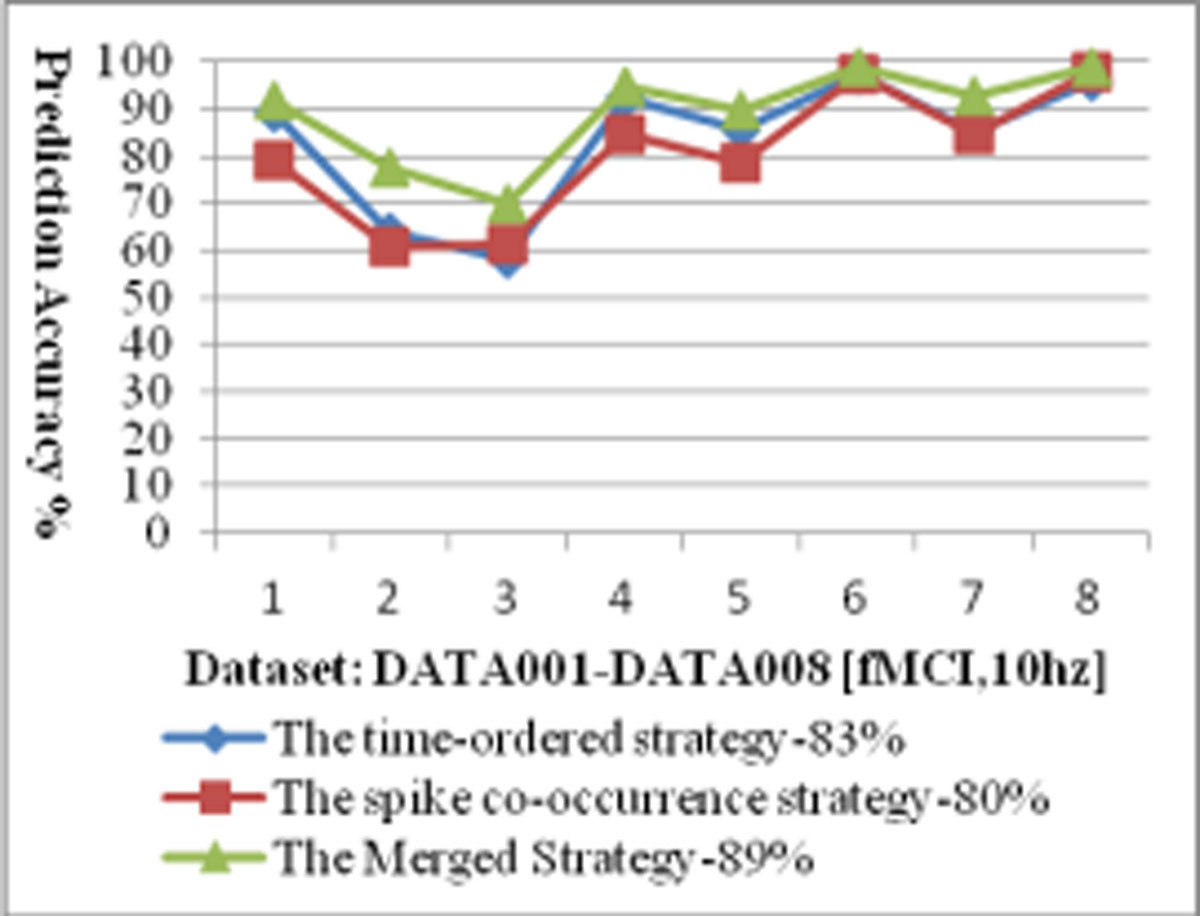
Neural Pathway Prediction Accuracy based on fMCI Multi-neuron Spike Train Dataset

There are several important observations and indications based on the prediction results. (1) Although the two prediction strategies seem entirely different, the neural pathways based on the two different strategies are very relevant (The correlation is significant with the Pearson correlation value 0.958). It indicates that although the proposed strategies are different, the results from the different two strategies do not have major conflicts, instead they are very consistent, and support each other. (2) The neural pathway prediction accuracy for the time-ordered strategy is 83%, and the accuracy for the spike co-occurrence strategy is 80%. When we group the two neural pathways together (denoted as the merged strategy), the prediction accuracy reaches 89% (Figure 1 shows the prediction accuracy for each of the dataset using the proposed strategies). This result indicates that better prediction can be made when the predicted pathways from the two strategies are combined together. (3) 27% of the possible connections among neurons are selected for the time-ordered strategy, while 25% of the connections are selected by the spike co-occurrence strategy. If the two results are grouped together, 32% of the possible connections are included. The results seem good, since the coverage is not high (and is consistent with the observation by using electro-microscopy techniques [3,4]), while the predicted accuracy for possible neural pathway reaches 89%.

The proposed method is validated on the data in which the distance of two neurons is within approximately 400um [5]. Whether the proposal is applicable when the distance goes further needs to be validated. In addition, our current result is based on fMCI data from rat brain slices. In our future work, we will investigate on the possibility of using the proposed method on fMCI imaging data from living animals.
